# A pathway approach to investigate the function and regulation of SREBPs

**DOI:** 10.1007/s12263-013-0342-x

**Published:** 2013-03-21

**Authors:** Sabine Daemen, Martina Kutmon, Chris T. Evelo

**Affiliations:** 1 Department of Bioinformatics, BiGCaT, Maastricht University, Maastricht, The Netherlands; 2 Netherlands Consortium for Systems Biology (NCSB), Amsterdam, The Netherlands

**Keywords:** SREBP, Cholesterol, Lipid metabolism, Signaling pathway, WikiPathways

## Abstract

The essential function of sterol regulatory element-binding proteins (SREBPs) in cellular lipid metabolism and homeostasis has been recognized for a long time, and the basic biological pathway involving SREBPs has been well described; however, a rapidly growing number of studies reveal the complex regulation of these SREBP transcription factors at multiple levels. This regulation allows the integration of signals of diverse pathways involving nutrients, contributing to cellular lipid and energy homeostasis. This review attempts to integrate this knowledge. The description of the SREBP pathway is Web-linked as it refers to the online version of the pathway on wikipathways.org, which is interactively linked to genomics databases and literature. This allows a more extensive study of the pathway through reviewing these links.

## Introduction

Sterol regulatory element-binding proteins (SREBPs) play an important role in the regulation of the intracellular cholesterol concentration and in overall lipid homeostasis. Since lipids and cholesterol are important components of cellular membranes and precursors for steroid hormones, bile salts, and essential signaling molecules, a tight regulation is vital. SREBPs provide a negative feedback mechanism by sensing the intracellular levels of cholesterol. SREBPs function as transcription factors, and upon activation, by low levels of cholesterol, they stimulate the expression of genes coding for proteins involved in the synthesis of cholesterol and fatty acids and in the uptake of lipoproteins (Brown and Goldstein 1997). The basic signaling pathway affected by SREBPs has been elucidated in great detail. However, regulation of SREBPs themselves is proven to be very complex. In the last few years, research has brought new insights regarding this regulation and the interaction with other nutrients and hormones that play a role in energy homeostasis. Recent studies also implicated the SREBP pathway to be important in the development of a range of pathological conditions, associated with obesity and the metabolic syndrome, like liver steatosis and hyperlipidemia (Moon et al. 2012). It has also been described that SREBP has a role in several physiological cellular processes not directly related with lipid homeostasis, like cell growth and innate immunity (Jeon and Osborne 2012). As the insight in the SREBP pathway becomes more and more complex, integration of the different aspects of this knowledge is vital. We will describe the SREBP protein and its isoforms, to continue with a description of the current view on the molecular basis of the SREBP pathway, its complex regulation and its physiological function. In this review, we are applying a pathway approach to investigate the function and regulation of the SREBP proteins in lipid-metabolism-related pathways.

## SREBP pathway

**Fig. 1:**
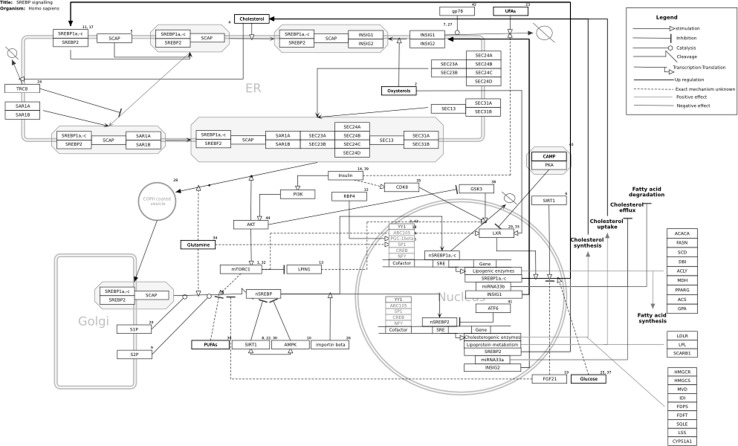
The SREBP pathway on WikiPathways

The description of the SREBP pathway in this review will especially focus on the role of the SREBP proteins in lipid-metabolism-related pathways. A graphical representation of the SREBP pathway (see Fig. 1) can be found on WikiPathways, a platform for community-based curation of biological pathways (Pico et al. 2008; Kelder et al. 2012). This pathway is a mammalian meta-pathway combining data from mouse, rat, and human studies. The description of the SREBP pathway will refer to this pathway representation on WikiPathways. The interactive pathway viewer on WikiPathways enables the user to zoom, pan, and browse to get detailed information on pathway elements in external databases and thereby allowing a more extensive study (Kelder et al. 2012). The pathway can be found at: http://wikipathways.org/index.php/Pathway:WP1982. The specific version we used for this review was: http://wikipathways.org/instance/WP1982_r59430. Various elements of the pathway (gene products, metabolites, interactions, and the pathway as a whole) are linked to literature references using Pubmed IDs. The gene products are among others linked to genomics databases like ENSEMBL (Flicek et al. 2011), Entrez (Maglott et al. 2011) and UniProt (Consortium 2012) and to databases providing information on biological function and the role in diseases, including Gene Ontology (Ashburner et al. 2000) and OMIM (Borate and Baxevanis 2009). The metabolites are linked to metabolite databases like HMDB (Wishart et al. 2013) and ChEBI (de Matos et al. 2010).

### The SREBP family

The SREBP family consists of three subtypes: SREBP-1a and SREBP-1c, which are the result of alternative promoter usage and transcription start sites in the SREBF1 gene, and SREBP-2. All three subtypes were identified by cDNA cloning (Yokoyama et al. 1993; Hua et al. 1993). SREBPs are transcription factors that bind to the sterol regulatory element (SRE) (Yokoyama et al. 1993). They are synthesized as endoplasmic reticulum (ER) membrane proteins. The SREBP protein consists of three domains: a N-terminal domain which has approximately 480 amino acids, in the middle a hydrophobic region of 80 amino acids containing two membrane-spanning domains and a C-terminal regulatory domain of 590 amino acids (Brown and Goldstein 1997). They are oriented in a hairpin fashion in the membranes of the ER and the nuclear envelope, in which the N-terminal and C-terminal project into the cytoplasm.

**Fig. 2:**
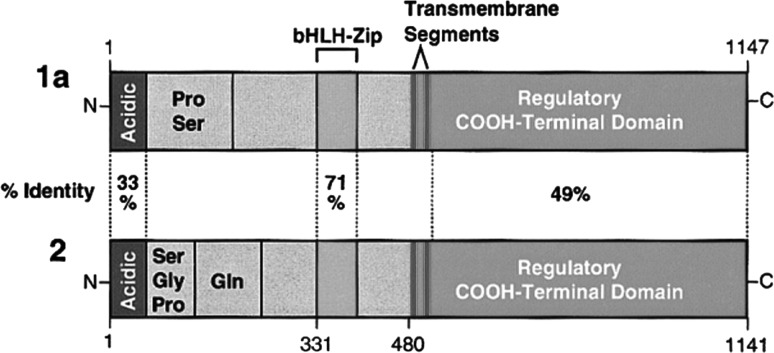
Protein domains of the SREBP isoforms. The structure of SREBP-1c is highly similar to SREBP-1a; SREBP-1c had a shorter transactivation domain in the N-terminus (Brown and Goldstein 1997)

The N-terminal domain is a basic-helix-loop-helix leucine zipper (bHLH-Zip). This domain is the functionally active portion of the SREBP and functions as the transcription factor. The N-terminal domain starts with an acidic domain that clusters acidic residues and functions as a transactivation domain. Deletion of this acidic domain converts SREBP-1 from an activator to an inhibitor of transcription (Sato et al. 1994). The acidic domain is followed by a region of which the function is unknown. In SREBP-1, this region is proline and serine rich, and in SREBP-2, this region is proline, serine, glutamine, and glycine rich. This region is then followed by the bHLH-Zip domain (Brown and Goldstein 1997). Figure 2 gives an overview of the different protein domains of the SREBP isoforms.

The high similarity among the N-terminal domains of the isoforms of SREBP results in the ability of all isoforms to activate all of the target genes identified so far, but with different efficiencies (Shimano 2001). Given that SREBP-1c has a shorter transactivation domain, this isoform is a less potent transcription factor than SREBP-1a and SREBP-2 (Shimano et al. 1997). Several in vivo studies obtained insight in the distinct roles of the SREBP isoforms. In transgenic mice that overexpress a truncated, active nuclear form of SREBP-2 in liver and adipose tissue, it was shown that SREBP-2 is a relatively selective activator of cholesterol synthesis, as opposed to fatty acid synthesis in these tissues (Horton et al. 1998). SREBP-1 knockout mice showed a significant decrease in mRNA coding for fatty acid synthesis enzymes. There was also a significant increase in cholesterol synthesis, but this was due to activation of SREBP-2, which compensated for the lack of SREBP-1 (Shimano et al. 1999). In general, SREBP-1 is relatively selective for lipogenic genes and SREBP-2 for cholesterogenic genes. This is due to differences among the SREBP isoforms in specificity for SREBP target promoters (Amemiya-Kudo et al. 2002; Pai et al. 1998).

### Activation of SREBP

Since SREBP is bound to the ER membrane, the N-terminal domain must be released before SREBP can activate its target genes in the nucleus. This requires a two-step proteolytic process, which takes place in the Golgi apparatus. Therefore, the SREBP is first transported to the Golgi apparatus. Important for the regulation of the cleavage of SREBP is another ER membrane-embedded protein named SREBP cleavage-activating protein (SCAP). In mice with a SCAP-deficient liver, no nuclear form of SREBP was found, and they showed an 80 % decrease in basal rates of cholesterol and fatty acid synthesis in the liver (Matsuda et al. 2001). SCAP has an N-terminal domain of 730 amino acids which has eight membrane-spanning regions separated by short hydrophilic loops, which include a sterol-sensing domain (SSD). This domain is similar to the sterol-sensing domain found in other proteins which interact with sterols: 3-hydroxy-3-methylglutaryl-Coenzyme A (HMG CoA) reductase, the Niemann-Pick disease type C1 protein and Patched (Hua et al. 1996). The C-terminal domain is a hydrophilic region of 546 amino acids containing 4 repeats of a tryptophan–aspartate repeat, WD. Both SREBP-1 and SREBP-2 form a complex with the SCAP protein on the ER membrane by binding of the WD region in SCAP to the C-terminal domain of SREBP. When there are enough sterols present in cells, cholesterol can bind directly to the sterol-sensing domain of SCAP, which then undergoes a conformational change. This conformation favors the binding of SCAP to another ER membrane protein named insulin-induced gene (Insig), which blocks translocation of the SREBP–SCAP complex to the Golgi apparatus, where the proteolytic activation takes place (Yang et al. 2002). This can be seen in the upper left corner of the pathway representation. The red arrow indicates the negative effect of cholesterol on SREBP stimulation by stimulating the binding of Insig to the SREBP–SCAP complex. Metabolites, like cholesterol, are indicated in blue boxes in the pathway representation.

There are two Insig isoforms, Insig-1 and Insig-2, which are both polytopic ER membrane proteins. They play an important role in the control of lipid synthesis, not only by binding to the SCAP protein. Insigs also bind to HMG-CoA reductase, which is the rate-limiting enzyme in the synthesis of cholesterol. The binding of Insig to HMG-CoA reductase induces the ubiquitination and proteolysis of this enzyme, whereas binding of Insig to SCAP leads to ER retention (Cao et al. 2007; Sever et al. 2003). The dual function of Insig in cholesterol metabolism is discussed in more detail in (Bengoechea-Alonso and Ericsson 2007). Insig-1 and Insig-2 demonstrate an amino acid identity of 59 % and are both embedded in the ER membrane by six membrane-spanning domains (Yabe et al. 2002). The regulation and the relative stability of the two isoforms differ. Insig-1 is itself a target of SREBP, whereas Insig-2a has been shown to be suppressed by insulin in hepatic cells (Yabe et al. 2003; Yellaturu et al. 2009b). The exact mechanism of the regulation of Insig by insulin remains unclear and is therefore visualized in the WikiPathways pathway using a dashed arrow. The Insig-1 protein is quite unstable and is degraded by the ubiquitin–proteasome pathway, whereas insig-2 is a relatively stable protein, which is constitutively expressed at low levels. In transgenic mice that overexpress human Insig-1 in the liver, the levels of all nuclear SREBPs (nSREBPs) were reduced, which shows that Insig inhibits SREBP processing (Engelking et al. 2004).

Upon sterol deprivation, the SREBP–SCAP complex dissociates from Insig and moves to the Golgi apparatus, a process that is discussed in more detail in the next section. Insig-1 is then ubiquitinated on lysines 156 and 158 by the membrane-bound ubiquitin ligase gp78. This ubiquitin ligase has a high affinity for Insig-1, and degradation of Insig-1 in a cholesterol-rich environment is probably prevented by binding competition between gp78 and SCAP (Lee et al. 2006). Insig-1 is subsequently degraded in proteasomes, providing a positive feedback mechanism on the activation of SREBP. nSREBPs activate the genes for cholesterol synthesis and uptake and stimulate the production of Insig-1. This upregulation can be seen in the pathway on the right in blue arrows. The new cholesterol and Insig-1 bind the SREBP–SCAP complex and the complex remains in the ER (Gong et al. 2006).

The cholesterol regulatory system is controlled not only by its end product cholesterol but also by oxysterols. Oxysterols are derivatives of cholesterol which have extra keto- or hydroxyl groups. Oxysterols were proven to regulate the interaction of SCAP and Insig, but they do so by a different mechanism than cholesterol. Cholesterol binds to SCAP, while oxysterols bind to Insigs. This induces SCAP to bind to Insig, which inhibits the movement of the SREBP–SCAP complex to the Golgi apparatus (Radhakrishnan et al. 2007). Oxysterols are also ligands of the nuclear liver X receptors (LXRs), which also play an important role in the cholesterol synthesis. Upon activation by oxysterols, LXR forms a heterodimer with the retinoid X receptors (RXRs) which binds to the LXR response element (LXRE) on target genes. An LXRE has been found in the proximal promoter region of the rat cytochrome P450 7A1 (CYP7A1) gene, which codes for an enzyme responsible for the rate-limiting step in the conversion of cholesterol to bile acids (Lehmann et al. 1997). However, in the human gene promotor of CYP7A1, the LXRE appears to be not conserved. In addition, in human primary hepatocyte cultures, it has been shown that activation of the LXR represses CYP7A1 expression, indicating a species-specific difference in the regulation of cholesterol homeostasis (Goodwin et al. 2003). In addition, LXRs have been implicated in the upregulation of genes involved in efflux of cholesterol from the cell, as ATP-binding cassette A1 (ABCA1). LXR/RXR can also bind the SREBP-1c promoter and induce SREBP-1 activation of fatty acid synthesis (Schultz et al. 2000).

### ER to Golgi transport

If there are not enough sterols present in the cell, the SREBP–SCAP complex moves to the Golgi apparatus through COPII-coated vesicles. The sorting of the complex in a COPII vesicle is depending on an amino acid sequence in the SCAP protein. SCAP has a long loop, which projects into the cytoplasm between the membrane-spanning helices 6 and 7. In this loop, the hexapeptide MELADL is found, which is required for the binding of the COPII proteins Sec23 and Sec24 to the SREBP–SCAP complex. Clustering of the SREBP–SCAP complex into a COPII vesicle is initiated by Sar1, a small GTPase that binds to the ER membrane GTP dependent. This binding is visualized on WikiPathways by a green arrow, which shows that this is the first step in the cascade toward activation of transcription by SREBPs. The binding of Sar1 initiates the binding of Sec23/24, which then recruits Sec13/31. This heterodimer forms the coat of the vesicle and the vesicle can bud from the ER membrane (Sun et al. 2005). Interaction of SCAP with Insig causes a conformational change in SCAP which inhibits the interaction of MEDADL with Sec23/24.

An ER membrane protein named ring finger protein 139, also called TRC8, shown in the upper right corner on WikiPathways, was identified as a regulator in the SREBP pathway. The protein contains a sterol-sensing domain (SSD) and a RING finger motif, which encodes for an E3 ubiquitin ligase. It is shown that the overexpressing of TRC8 inhibits SREBP-2 processing. TRC8 is capable of binding both SREBP-2 and SCAP and a TRC8–SREBP–SCAP complex is formed. This inhibits the binding of SCAP to Sec23/24 and blocks transport of the SREBP–SCAP to the Golgi apparatus. The TRC8 protein in itself is highly unstable because of self-ubiquitination, which leads to degradation. When cells were cultured with a lipoprotein-deficient serum, the TRC8 protein became stable (Irisawa et al. 2009). It is thus likely that when the SSD senses a decline in lipoprotein, it will downregulate the E3 ligase activity. It could provide a brake on the SREBP processing in conditions of sterol depletion, preventing too much processing of SREBP (Sato 2010).

### Proteolytic cleavage

After fusion of the COPII vesicle with the Golgi apparatus, the N-terminal of the SREBPs is released by intramembrane proteolysis. The processing of SREBP–nSREBP is shown in the bottom left corner on WikiPathways. The process is executed by two proteases, membrane-bound transcription factor peptidase site 1, or Site-1 protease (S1P), and Site-2 protease (S2P). The process is initiated when S1P, a membrane-bound serine protease, cleaves the leucine–serine bond in the sequence RSVLS within the luminal loop of SREBP (Duncan et al. 1997). This separates the two membrane-spanning segments. The next step is cleavage by S2P, which hydrolyzes a leucine–cysteine bond in the sequence DRSRILLC. This sequence lies within the N-terminal membrane-spanning domain, and cleavage occurs in three residues in this domain (Duncan et al. 1998). The result is that the N-terminal domain is released from the SREBP and functions as an active nSREBP, which migrates to the nucleus to activate target genes (Brown and Goldstein 1999). It has been proposed that the cleavage of S1P is required for the cleavage of S2P, because the separation of SREBP into two halves causes a conformational change in the first membrane-spanning domain which allows S2P to be exposed to its target sequence, thus favoring the cleavage of S2P (Ye et al. 2000). In addition, it has been demonstrated that caspase 3, a cysteine protease that is involved in the induction of apoptosis, releases mature SREBP from the ER membrane, probably in a sterol-independent manner (Higgins and Ioannou 2001).

### SREBP target

The nSREBPs released during the cleavage reaction travel into the nucleus. This nuclear transport is mediated by karyopherin (importin) beta, which interacts with the bHLH-Zip motif (Nagoshi et al. 1999). Important genes involved in lipid metabolism that are activated by SREBP are listed individually on WikiPathways. Besides activating these target genes, SREBPs also induce transcription of the SREBP gene itself, which contains a SRE, and thus stimulate production of new SREBPs and provide a positive feedback loop. Although SREBPs mainly activate target genes, genes with a SRE sequence have been reported which are repressed by SREBPs. These genes are, for example, microsomal triglyceride transfer protein (MTTP) (Sato et al. 1999) and caveolin (Bist et al. 1997). Since SREBP is active in cases of cholesterol depletion, it is likely that SREBPs repress these genes, which are involved in the efflux of cholesterol and the secretion of lipoproteins (Shimano 2001). The inhibition of genes by SREBP could be due to an indirect effect, namely through activation of repressors. For example, in human myotubes, it has been shown that the transcriptional repressor genes BHLHB2 and BHLHB3 are SREBP-1 target genes, negatively regulating skeletal muscle development (Lecomte et al. 2010).

Activation of the target genes by SREBP requires several cofactors. Usually, nuclear transcription factor Y (NF-Y), Sp1 transcription factor, and CREB-binding protein (CBP) act as cofactors for SREBP. Binding sites for these factors are often found in the SREBP target gene promoters and they are involved in the assembly of the transcription machinery (Bennett and Osborne 2000). The activator recruited-cofactor (ARC)-mediated co-activator complex, a large complex that associates with RNA polymerase II, has also been found to interact with SREBPs. They have been shown to use ARC105 to activate target genes (Yang et al. 2006). The peroxisome proliferator-activated receptor-γ coactivator-1 (PGC-1) family functions as important regulators of lipid metabolism. PCG-1β has been found to interact with SREBPs and works as a transcriptional co-activator in the transcription of lipogenic genes (Lin et al. 2005).

Another transcription factor named the YY1 transcription factor seems to be negatively involved in the regulation of SREBP target gene activation. It is shown that the promoters of the HMG-CoA synthase, farnesyl diphosphate (FPP) synthase, and the low-density lipoprotein (LDL) receptor contain YY1 binding sites. YY1 seems to repress SREBP activation by the displacement of NP-Y from the promoter (Ericsson et al. 1999). Other studies suggest YY1 acts by inhibiting the interaction between Sp1 and SREBP (Bennett et al. 1999). The physiological role of YY1, however, is yet to be identified. On WikiPathways, the cofactors are drawn on a cofactor-binding site in the promoter of the target genes. The green colored boxes show activators, whereas the red colored boxes represent repressors.

Interestingly, the SREBF-1 and SREBF-2 gene loci contain, respectively, miR33b in intron 17 and miR33a in intron 16. The mature microRNAs differ in only two nucleotides, but are thought to have a largely overlapping target gene set (Davalos et al. 2011; Rottiers and Naar 2012). These microRNAs appear to work synergistically with SREBP in increasing fatty acid synthesis and cholesterol synthesis and uptake (Rayner et al. 2010; Horie et al. 2010; Gerin et al. 2010). Interestingly, rodents lack the miR33b gene in the SREBF-1 gene (Rayner et al. 2010). Both miR33a and miRNA33b seem to inhibit the expression of genes involved in fatty acid degradation, e.g., carnitine *O*-octanoyltransferase (CROT), and genes that negatively regulate fat production, e.g., insulin receptor substrate 2 (IRS2) (Rottiers and Naar 2012). In addition, they also repress expression of ATP-binding cassette transporter A1 (ABCA1), which normally promotes the efflux of cholesterol from cells to apolipoprotein A1 (APOA1), leading to high-density lipoprotein (HDL) formation (Horie et al. 2010).

### Regulation of the SREBP pathway

Expression and processing of the isoforms of SREBP in vivo was found to be very complex. The SREBP pathway is not just regulated on cell level by the intracellular level of cholesterol, but it can be affected by the nutritional and hormonal status of the body as a whole.

Several studies provided a link between insulin, glucose, and SREBPs. It is known that glucose and insulin stimulate fatty acid synthesis through activation of hepatic lipogenic genes. It has been recognized the PI3K/AKT pathway plays an important role in the regulation of SREBP by insulin. A range of studies has been done on exploring the effect of the PI3K/AKT pathway on SREBP, and effects on transcription, activity, processing, and stability have been found (Krycer et al. 2010). A possible mechanism is that insulin increases the migration of the SREBP–SCAP complex from ER to Golgi. Insulin stimulates Akt/PKB-dependent phosphorylation of serine and threonine residues of SREBP-1c. This leads to an increased affinity of the SREBP–SCAP complex for de COPII proteins, Sar1 and Sec23/24, and a decreased affinity for Insig, which retains the SREBP–SCAP complex in the ER membrane (Yellaturu et al. 2009a). It has also been shown that insulin enhances processing of SREBP-1c in hepatic cells by stimulation of the degradation of Insig-2a mRNA, reducing Insig-2a protein levels (Yellaturu et al. 2009b). The PI3K/AKT pathway inhibits glycogen synthase kinase 3 (GSK3) through phosphorylation. It has been proposed that this diminishes degradation of mature SREBP-1, since GSK3 has been shown to promote ubiquitination and proteasomal degradation of SREBP-1 through a phosphorylation cascade; GSK3 phosphorylates SREBP-1 at Ser-434, whereby it increases its own affinity for Ser-430 and Thr-426 in SREBP-1, leading to GSK-3-dependent phosphorylation of these sites and a binding site for the ubiquitin ligase Fbw7 (Bengoechea-Alonso and Ericsson 2009). One of the major downstream regulators of the PI3K/AKT pathway is the mammalian target of rapamycin (mTOR). In the past, it has been shown that the mTOR complex-1 (mTORC1) positively regulates the processing of SREBP-1. It was thought this activation was mediated by the ribosomal protein S6 kinase (RPS6K2), which is phosphorylated by mTORC1 (Duvel et al. 2010). It has recently been shown that insulin-mediated stimulation of SREBP-1c processing required mTOR, studied in a hepatic system in which the effect of insulin on SREBP-1c processing could be dissected from the effect of insulin on SREBP-1c transcription, described below. This stimulation of SREBP processing by insulin could be inhibited by using an inhibitor of p70 ribosomal S6K, leading to an increase in nSREBP-1c, which was more likely due to an increased production of nSREBP-1c then decreased degradation. The mechanisms by which S6K can lead to increase in nSREBP-1c require further investigation (Owen et al. 2012; Quinn and Birnbaum 2012).

In addition, it has been suggested that the regulation of SREBP-1 is achieved by the regulation of the nuclear entry of phosphatidate phosphatase lipin 1 by mTORC1. Lipins are involved in triacylglycerol biosynthesis and have a second function as transcriptional co-activators. Lipins are sequestered in the cytosol in a hyper-phosphorylated state, and phosphorylation is induced by mTORC1. Loss of mTORC1-mediated lipin 1 phosphorylation promotes the nuclear entry of lipin 1, and this promotes downregulation of nSREBP, of which the exact mechanism is unknown (Peterson et al. 2011).

Also, insulin can increase basal transcription of the SREBP-1c gene. The liver X receptor has been reported to have a central role in this insulin-mediated activation of SREBP-1c transcription. In the mouse promoter of SREBP-1c, two LXR elements have been found. In rat primary hepatocytes, it was shown that disruption of both LXREs blunts the effect of insulin on transcription of SREBP-1c (Chen et al. 2004). In contrast, another study did not find a major involvement of the LXREs in the response to insulin, but insulin requires the presence of SRE in the SREBF-1 promoter and enhanced the binding of SREBP-1 to its own promoter. However, it should be noticed that this study made use of a different system based on HEK293 cells (Dif et al. 2006). cAMP, which can be activated by glucagon, and the cAMP-dependent kinase, protein kinase A (PKA) have been shown to suppress SREBP-1c transcription by phosphorylation of LXR, which inhibits the DNA binding activity by inhibiting LXR/RXR dimerization, decreases recruitment of a coactivator, and enhances the recruitment of a corepressor (Yamamoto et al. 2007). In addition, it has been shown in HepG2 cells that PKA can phosphorylate SREBP-1a at Ser338, which reduces DNA binding of SREBP-1c (Lu and Shyy 2006). These results indicate a role for the cAMP/PKA pathway in mediating SREBP-1 and hepatic lipogenesis.

It has been shown that the increase in SREBP-1 expression stimulated by insulin can be inhibited by wortmannin and rapamycin, indicating the PI3K-mTORC1 pathway is involved. In contrast to the stimulation of SREBP-1c processing by insulin, the increase in SREBP-1 expression by insulin could not be blocked by inhibiting S6K. This suggests that the regulation of SREBP-1c by insulin bifurcates downstream of mTORC1, with one arm controlling the processing of SREBP-1c and the other the gene expression (Owen et al. 2012; Quinn and Birnbaum 2012). Furthermore, it has been shown that upstream of this in the liver, by using liver-specific rictor knockout mice, insulin stimulates mTOR complex-2 (mTORC2), which phosphorylated Akt at serine 473, leading to SREBP-1c activation (Hagiwara et al. 2012). Other studies showed that a glucose-dependent increase in SREBP-1c protein, shown in the lower right corner of the pathway, was due to an increase in SREBP-1 mRNA, suggesting that glucose regulates the expression of SREBP-1c at transcriptional level (Hasty et al. 2000). In a human renal proximal tubular cell line, it was shown the glucose-dependent activation of SREBP was potentially mediated through the PI3K/AKT pathway (Hao et al. 2011). SREBP-2 levels remained unchanged when treated with insulin and glucose in the liver. That insulin only stimulates hepatic SREBP-1, and not SREBP-2, matches the fact that insulin and SREBP-1 have both been shown to induce lipogenesis. However, in the brain, it has been shown that in insulin-deficient diabetic mice, there is a reduction in the expression of SREBP-2, suggesting that in the brain, insulin upregulates SREBP-2 expression (Suzuki et al. 2010). A complete picture of the regulation of SREBP by insulin and glucose requires additional studies. In addition, cyclin-dependent kinase 8 (CDK8) and its regulatory partner cyclin C (CycC), which are part of the coactivator mediator complexes in mammalian cells, have been identified as regulators of de novo lipogenesis in *Drosophila*. Site-specific phosphorylation of nuclear SREBP-1c by CDK8 results in an enhanced ubiquitination and degradation of nSREBP-1c. Insulin and feeding decreased the levels of CDK2 and CycC and enhanced the levels SREBP-1c, indicating CDK8–CycC acts downstream of insulin in the regulation of de novo lipogenesis (Zhao et al. 2012).

A crosstalk between SREBP and carbohydrate responsive element-binding protein (ChREBP) has been found. These transcription factors appear to work synergistically to regulate glycolytic and lipogenic gene expression. The phosphorylation of glucose to glucose-6-phosphate by hepatic glucokinase (GK) was found to be essential in the induction of glycolytic and lipogenic genes (Dentin et al. 2004). SREs have been found in the GK promoter, which is an indication that SREBP can activate GK expression after activation by insulin. In the presence of high glucose, xylulose 5-phosphate (X5P) can be formed, which can activate protein phosphatase 2A (PP2A). This phosphatase can dephosphorylate ChREBP, leading to nuclear translocation of this transcription factor, where it binds to carbohydrate response element (ChRE) in the promoter of glycolytic and lipogenic genes. In addition, SREBP-1c can stimulate glycolytic and lipogenic gene transcription after stimulation by insulin. Thus, in the presence of high glucose and insulin, ChREBP and SREBP can work synergistically to activate glycolytic and lipogenic genes (Dentin et al. 2005).

Activating transcription factor-6 (ATF6) has also been found to interact with SREBP-2. ATF6 is also an ER membrane-bound transcription factor, which upon stimulation is translocated from ER to Golgi, where proteolytic cleavage by S1P and S2P occurs (Chen et al. 2002). ATF6 is stimulated by the accumulation of misfolded or unfolded proteins and this ER stress could be caused by glucose deprivation. The cleaved ATF6 translocates to the nucleus and binds to nSREBP-2 bound to target genes promoters. The nuclear ATF6 recruits histone deacetylase 1 (HDAC1), which downregulates SREBP-2 gene expression. The physiological relevance could be that when glucose is depleted, lipogenesis and cholesterogenesis are downregulated to save energy (Zeng et al. 2004).

Alternative regulators of the SREBP pathway are polyunsaturated fatty acids (PUFAs), another example of how diet influences the activation of SREBP. PUFAs have been known as negative regulators of hepatic lipogenesis and have an inhibitory effect on the SREBP pathway. PUFAs appear to suppress the proteolytic processing of SREBP-1c. Suppression of the proteolytic processing of SREBP in turn leads to a decrease in SREBP-1c transcription through lowering SREBP-1c binding to SRE on its own promoter. The exact molecular mechanism underlying this suppression still remains unknown, which is shown by the dashed arrow in the pathway. PUFAs do not seem to affect the functioning of SREBP-2 (Takeuchi et al. 2010). There are several reports suggesting LXR is involved in transcriptional regulation of SREBPs by PUFA (Ou et al. 2001; Yoshikawa et al. 2002). However, several other studies did not find an involvement of LXR in the regulation by PUFA, which could be due to different study systems being used (Takeuchi et al. 2010; Deng et al. 2002). In addition, it has been shown that unsaturated fatty acids inhibit proteosomal degradation of Insig-1. Membrane proteins of the ER can be degraded by the ubiquitination-proteasome system in a process called ER-associated degradation (ERAD). In this process, valosin-containing protein (VCP) extracts ubiquitinated proteins from the membrane making the proteins accessible for degradation in the proteasome. Another protein, named Ubxd8, recruits VCP to Insig-1. Unsaturated fatty acids (UFAs) appear to block to interaction between Ubxd8 and VCP, thereby inhibiting the extraction of Insig-1 from the membrane (Lee et al. 2008).

Recent findings suggest the amino acid glutamine is also involved the regulation of the gene expression and processing of SREBPs, suggesting another link between amino acid metabolism and lipid metabolism. Glutamine seems to increase mRNA levels of several SREBP targets. Glutamine aids in the gene expression of SREBP-1 by increasing the binding of the transcription factor Sp1 to the SREBP-1a promoter. Glutamine also increases the processing of the SREBP protein, presumably by stimulating the transport of the SREBP–SCAP complex from ER to the Golgi apparatus (Inoue et al. 2011).

The NAD^+^-dependent deacetylase SIRT1 has been shown to directly deacetylate SREBP-1c, leading to a decreased stability of the protein and a reduced association of SREBP-1c with its target genes (Ponugoti et al. 2010). Furthermore, SIRT1 has been shown to downregulate target gene expression by SREBP-1c in vivo under fasting conditions (Walker et al. 2010). Whereas these studied focuses on the liver, recently, it has been shown that SIRT1 also regulated SREBP-1c expression in skeletal muscle. Interestingly, the effect of SIRT1 on SREBP-1c expression was completely abolished when the LXR response elements in the SREBF-1 promoter were deleted, which suggest SIRT1 regulates SREBP-1c expression in muscle by deacetylation of LXR transcription factors (Defour et al. 2012). In addition, AMP-activated protein kinase (AMPK) has been shown to directly phosphorylate SREBP-1c and thereby directly inhibit SREBP-1c processing and translocation to the nucleus in the liver (Li et al. 2011b). Interestingly, there is evidence that AMPK and SIRT1 stimulate each other and share targets (Ruderman et al. 2010).

A link has also been found between fibroblast growth factor 21 (FGF21) and SREBP-1c in hepatocytes. FGF21 has been identified as a regulator of energy homeostasis, glucose, and lipid metabolism. However, little is known about the regulation or activity of this FGF. It was found that FGF21 downregulated the transcription of SREBP-1c, but the processing of SREBP-1c to its mature form was also diminished. Interestingly, it was found that SREBP-1c could also inhibit FGF21 expression. Molecular mechanisms and biological relevance of this link remain unclear for the time being (Zhang et al. 2011).

Recently, the role of retinol binding protein 4 (RBP4) in lipogenesis has been explored. In HepG2 cells, human RBP4 induces an increase in mature SREBP-1 and its nuclear translocation, which was also confirmed in an in vivo experiment. In addition, treatment of HepG2 cells with RBP4 leads to a strong upregulation of the expression and protein levels of PCG-1β. This suggests that RBP4 induces SREBP-1 activation through induction of PCG-1β, leading to an increase in hepatic lipogenesis (Xia et al. 2013). Earlier, it was already reported that retinoic acid and retinal can synergize with insulin to induce the expression of SREBP-1c in primary rat hepatocytes. This was mediated via the retinoid X receptor. This indicated a role of retinol in regulating hepatic lipogenesis (Li et al. 2011a).

### Other roles of SREBP

The important function of SREBP in lipid metabolism led to these proteins being involved in a variety of pathological conditions related to lipid metabolism, as steatosis and hyperlipidemia (Moon et al. 2012). However, several other functions of SREBP, not directly related to lipid metabolism, have emerged recently. SREBPs have been found to regulate several cellular processes, including autophagy, phagocytosis, membrane biogenesis, immunity, hypoxia, and the cell cycle. SREBP-2 has been found to occupy promoters of genes that are involved in mediating autophagy and knockdown of SREBP-2 decreases autophagosome formation in cholesterol-depleted cells, indicating a role for SREBP-2 in autophagy (Seo et al. 2011). Phagocytosis occurs especially within the phagocytic cells of the innate immune system to engulf exogenous particles. Phagocytosis can promote membrane biogenesis via the activation of SREBP-1a and SREBP-2 (Castoreno et al. 2005). In addition, it has been reported that bacterial pore-forming toxins can trigger cleavage and activation of SREBP-1 and SREBP-2, probably through caspase-1, which could aid in membrane repair (Gurcel et al. 2006). Furthermore, it has been found that SREBP-1a can induce expression of the anti-apoptotic gene Api6 when toxin is present, which promotes cell survival (Im and Osborne 2012). In fission yeast, it was found that SREBP homologs stimulated transcription of genes that are involved in adaption to hypoxia in response to low oxygen levels (Hughes et al. 2005). Several reports found SREBP to be involved in cell cycle control. nSREBP-1 appears to be hyperphosphorylated by cyclin-dependent kinase (CDK)1/Cyclin B during mitosis, which stabilizes nSREBP. Furthermore, inactivation of SREBP-1 arrested the cells in the G1 phase of the cell cycle (Bengoechea-Alonso and Ericsson 2006). In addition, expression of the major CDK inhibitor p21 was found to be induced by SREBP-1 (Inoue et al. 2005). Interestingly, miR33, located in the SREBP gene locus, also appears to be involved in the regulation of the cell cycle. miR33 inhibits CDK6 and cyclin D1 and thus reduces cell cycle progression, with overactivation of miR33 even leading to a cell cycle arrest in the G1 phase (Cirera-Salinas et al. 2012). The roles of SREBP beyond lipid metabolism have been reviewed in more detail in recent reviews (Jeon and Osborne 2012; Shao and Espenshade 2012).

## Conclusion

We previously described how literature review can be used to obtain highly curated pathways for biological processes (Adriaens et al. 2008; Jennen et al. 2010), which can be used for data analysis in PathVisio (van Iersel et al. 2008). The new interactive browsing functionality of WikiPathways now allows the pathways themselves to be used as interactive means to study relevant literature and database information on the reactions and entities involved, their known roles in biology and disease, relevant genetic variation, chemical properties, etcetera. The SREBP pathway on WikiPathways described here is an example that makes full use of this functionality.

The basic pathway of SREBP signaling has been well described. When sterol levels are high, Insig retains the SREBP–SCAP complex within the ER membrane. In case of sterol depletion, the SREBP–SCAP complex interacts with COPII proteins and migrates in COPII vesicles to the Golgi apparatus. In the Golgi apparatus, SREBP is cleaved and active nuclear SREBP is released. This nSREBP migrates to the nucleus to activate target genes involved in lipid metabolism. However, the regulation of the pathway proves to be very complex and there are still many unanswered questions, especially regarding target genes and regulation. Increasingly, links are being found between the SREBP pathway and other regulators of lipid, protein, and carbohydrate metabolism and overall energy homeostasis: PUFAs are an example how diet influences the SREBP pathway, the link found between glutamine and SREBP suggests another link between amino acid metabolism and lipid metabolism, the interaction of ATF6 and SREBP-2 could imply that the synthesis of cholesterol is slowed in case of energy stress through SREBP-2 inhibition. Especially important to recognize are the links between insulin, glucose, and SREBP, suggesting an important role for SREBP in the pathology of current diseases, as obesity and the metabolic syndrome. Combining and integrating the growing knowledge on the SREBP pathway is essential, in which biological pathway creation and curation can play a major role.
